# 

**Published:** 1899-03-04

**Authors:** 


					THE HOSPITAL, "The standard of highest purity."?Lancet, Marofi 4th, 1899,
CADBURY'S mA.'EY 19* CADBURY'S
COCOA ft JOl jPU COCOA
ABSOLUTELY PURE. NO DRUGS OR ALKALIES USED.
' * ?iJU?LiA?aoirv Wis term IhfirmjlAX
No. 049. Vol. XXV. [^^"3 SatTOOAY,
March 4th, 1899.
[SabBoriptionTerms.] pBiICI; TWOPENCE.

				

